# Taking the Road Less Travelled: A Case Report of Distal Splenic Artery Embolisation via the Pancreaticoduodenal Arcade in Splenic Trauma With Celiac Artery Stenosis

**DOI:** 10.7759/cureus.64094

**Published:** 2024-07-08

**Authors:** Harsha M T., Mohit Singh, Pankaj Sharma, Samanvitha H.

**Affiliations:** 1 Interventional Radiology, All India Institute of Medical Sciences, Rishikesh, Rishikesh, IND; 2 Radiodiagnosis, Postgraduate Institute of Medical Education and Research, Chandigarh, Chandigarh, IND; 3 Diagnostic Radiology, Bangalore Medical College and Research Institute, Bengaluru, IND

**Keywords:** splenic artery embolisation, pancreaticoduodenal arcade, collateral pathway, splenic injury, celiac axis stenosis, case report

## Abstract

Celiac axis stenosis (CAS) is one of the most prevalent splanchnic arterial pathologies. It seldom results in clinically severe ischemic bowel disease because of the rich collateral circulation from the superior mesenteric artery. Knowledge about the collaterals in celiac artery stenosis guides various interventional procedures. Here, we describe a case of a 19-year-old female with American Association for the Surgery of Trauma (AAST) grade IV splenic injury found to have CAS. Distal splenic artery embolisation was performed via the collateral pathway through the pancreaticoduodenal arcade.

## Introduction

The celiac axis supplies blood to vital organs of the foregut. A high prevalence of asymptomatic celiac artery and/or superior mesenteric artery (SMA) stenosis (stenosis of 50% or more) of 30.4% was seen in patients with peripheral vascular disease [1]. According to Roobottom and Dubbins, the prevalence of significant celiac axis stenosis (CAS) in asymptomatic patients was 3% (<65 years) and 18% (>65 years) [2]. Park et al. reported a prevalence of significant CAS in 7.3% of asymptomatic population [3]. Extensive collateral circulation from the SMA prevents severe ischemic bowel disease in CAS. The most commonly injured solid organ in blunt abdominal trauma is the spleen [4]. The immunological role of the spleen necessitates splenic preservation procedures. Endovascular splenic artery embolisation (SAE) is the current treatment of choice for the American Association for the Surgery of Trauma (AAST) grades III-V injuries in hemodynamically stable patients. We present a case of distal SAE via pancreaticoduodenal arcade in splenic trauma with co-existing CAS.

## Case presentation

A 19-year-old female with a road traffic accident presented to the trauma centre. She had no co-morbidities or significant history. On admission, her vitals were stable. The examination revealed a tender abdomen.

**Figure 1:**
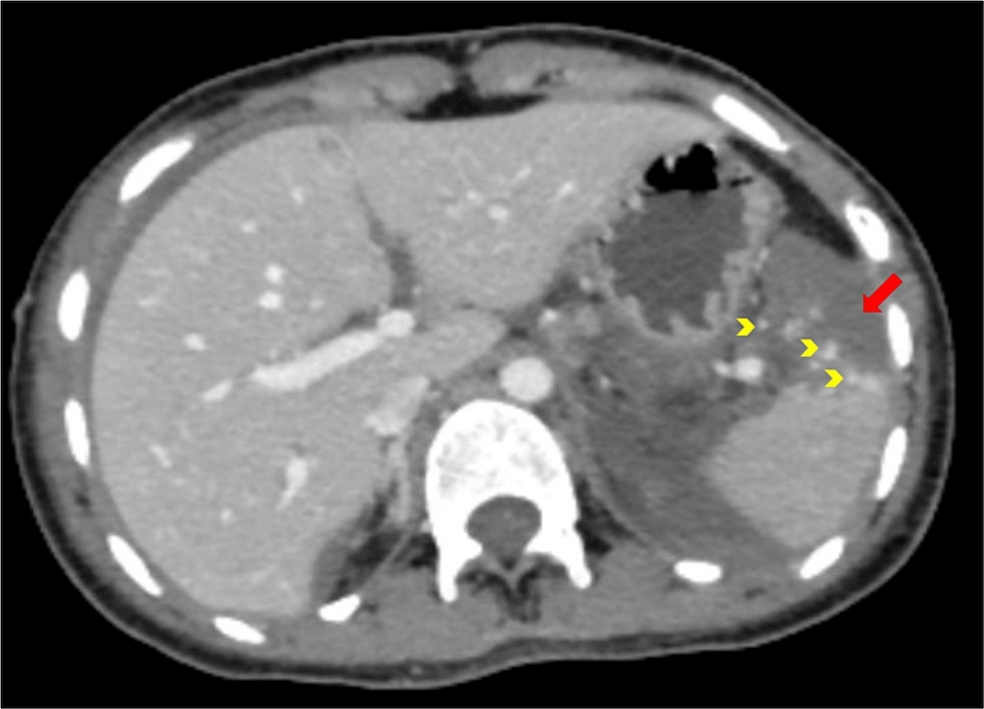
AAST grade IV splenic injury Contrast-enhanced CT of the abdomen in the portal venous phase shows areas of devascularisation involving >25% splenic parenchyma (red arrow) along with focal hyperdense traumatic pseudoaneurysms (yellow arrowheads) in the interpolar region and lower pole, as well as subcapsular and perisplenic hematoma. These findings are suggestive of AAST grade IV splenic injury. AAST: American Association for the Surgery of Trauma

**Figure 2:**
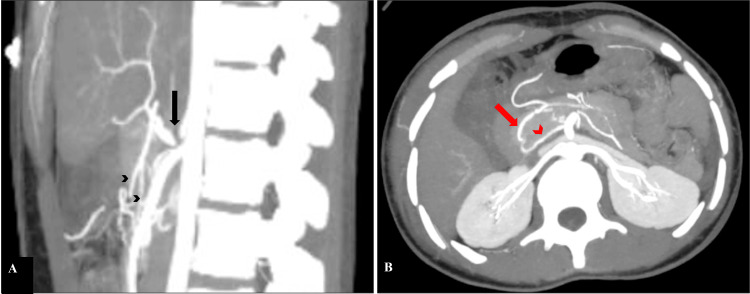
Celiac artery stenosis and the collateral pancreaticoduodenal arcades Contrast-enhanced CT scan of the abdomen, sagittal (A) and axial (B) sections in maximum intensity projection (MIP), shows focal interruption (black arrow) of the contrast column near the Ostia of the celiac trunk with normal opacification of the distal celiac trunk and its branches (black arrowheads). Image B depicts the course of pancreaticoduodenal arcades. The anterior arcade (red arrow) is anteriorly and laterally placed, and runs in the pancreaticoduodenal groove. The posterior arcade (red arrowhead) is posteriorly and medially located.

Contrast-enhanced computed tomography (CECT) of the abdomen revealed extensive splenic lacerations with areas of devascularisation involving >25% splenic parenchyma with focal hyperdense traumatic pseudoaneurysms suggesting AAST grade IV splenic injury (Figure 1) with moderate hemoperitoneum. There was a focal interruption of contrast at the Ostia with normal opacification of the celiac trunk and its branches (Figure 2). Our case neither showed extrinsic compression of the celiac trunk by the median arcuate ligament (MAL) nor atherosclerotic plaque. On admission, her haemoglobin was 11.0 g/dL. After 12 hours, it dropped to 7.5 g/dL with a blood pressure of ~88/66 mmHg, indicating an emergency SAE.

**Figure 3:**
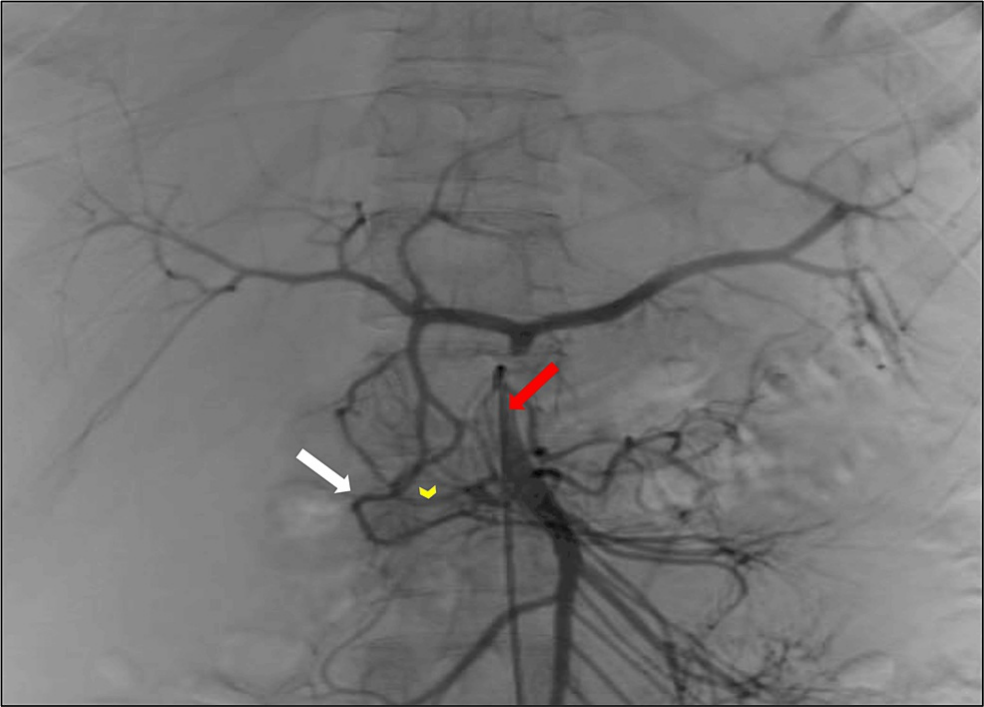
The collateral pathway - pancreaticoduodenal arcades Superior mesenteric angiogram with C2 catheter (red arrow) shows anterior (white arrow) and posterior (yellow arrowhead) pancreaticoduodenal arcades arising separately from SMA. Retrograde contrast filling of gastroduodenal, common hepatic artery and celiac trunk via the pancreaticoduodenal arcades is seen. The splenic artery is also opacified. SMA: superior mesenteric artery

**Figure 4:**
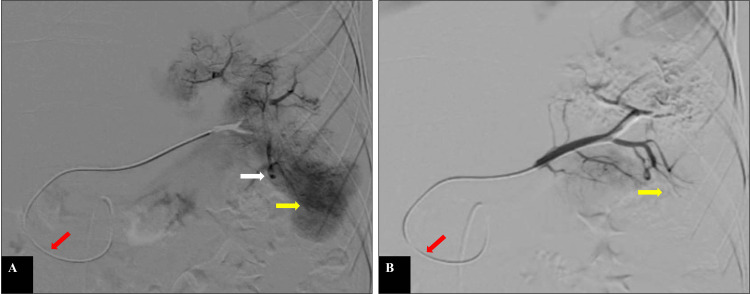
Selective splenic angiogram before and after selective distal embolisation Selective splenic angiogram is done with a 2.7 Fr microcatheter via the anterior pancreaticoduodenal arcade (red arrow) before (A) and after (B) selective distal embolisation of the interpolar region and lower pole with gel foam. Image A shows an abnormal parenchymal blush (yellow arrow) in the interpolar region and a lower pole with pseudoaneurysm (white arrow) in the interpolar region of the spleen. Image B shows the disappearance of abnormal parenchymal blush after selective distal embolisation of the interpolar region and lower pole with gel foam (yellow arrow).

The celiac trunk could not be hooked with a C2 catheter due to complete ostial occlusion. Therefore, SMA was hooked with a C2 catheter. The SMA angiogram showed collateral circulation between the celiac trunk and SMA, identified as anterior and posterior pancreaticoduodenal arcades (Figure 3). Two inferior pancreaticoduodenal artery branches formed the SMA counterpart. Contrast filled the celiac trunk and its branches in a retrograde manner. Using a 0.021 inch, 150cm J tip Terumo guidewire (Terumo Corporation, Tokyo, Japan), SMA was catheterized, followed by catheterization of the collateral pathway - specifically the anterior pancreaticoduodenal arcade, the common hepatic artery and the celiac trunk - using 2.7 Fr microcatheter in a retrograde direction to gain access into the splenic artery. The splenic angiogram (Figure 4A) revealed abnormal contrast blush and small pseudoaneurysms in the interpolar region and inferior pole of the spleen. Selective distal embolisation was done using gel foam. The check angiogram did not reveal any contrast leak (Figure 4B). The patient withstood the procedure well and improved clinically.

## Discussion

CAS can be due to intrinsic (atherosclerosis or dissection) or extrinsic (MAL syndrome or malignancy) etiologies [5]. According to Park et al. [3], inconclusive etiology was seen in 10 out of 400 patients, seven of whom fell into the younger age group. Despite being a common occlusive vascular disease, CAS seldom results in clinically severe ischemic bowel disease because of the extensive collateral circulation from SMA [6].

The most common collateral vessels from SMA in CAS cases are the pancreaticoduodenal arcade and the dorsal pancreatic artery. Knowledge about this collateral circulation is essential for interventional procedures, surgical procedures for periampullary carcinoma, and liver transplantation [7].

In our case, the pancreaticoduodenal arcade is the sole collateral pathway between SMA and the celiac axis. The anterior superior pancreaticoduodenal artery, one of the terminal branches of the gastroduodenal artery, and the inferior pancreaticoduodenal artery of SMA form the anterior pancreaticoduodenal arcade. The posterior arcade is formed between the retroduodenal artery (aka posterior superior pancreaticoduodenal artery) and the inferior pancreaticoduodenal artery of SMA.

AAST grades III-V splenic injuries or demonstration of pseudoaneurysms, traumatic arteriovenous fistulas, or extravasation on CT scans indicate an endovascular SAE [8]. The technique of SAE is celiac angiography to evaluate the anatomy of the splenic artery and collateral pathways, followed by microcatheterisation and embolisation of the splenic artery or its branches via the celiac artery. In cases of CAS, selective catheterisation of the collateral circulation is the first and most important step in interventional procedures. Expertise is required to catheterise a highly stenotic celiac artery or other collateral channels without injuring the artery, and the procedure may occasionally need to be stopped [9].

## Conclusions

Apart from anatomical variations of the celiac axis, the knowledge of various collateral pathways between the celiac axis and SMA in the case of CAS is pivotal and helps in planning interventional procedures. Selective catheterisation of the collateral pathways or highly stenotic celiac arteries is a crucial step and needs expertise.
